# Miniature probe for mapping mechanical properties of vascular lesions using acoustic radiation force optical coherence elastography

**DOI:** 10.1038/s41598-017-05077-7

**Published:** 2017-07-05

**Authors:** Yueqiao Qu, Teng Ma, Youmin He, Mingyue Yu, Jiang Zhu, Yusi Miao, Cuixia Dai, Pranav Patel, K. Kirk Shung, Qifa Zhou, Zhongping Chen

**Affiliations:** 1 Beckman Laser Institute, University of California, Irvine, 1002 Health Sciences Rd., Irvine, CA 92617 USA; 2 Department of Biomedical Engineering, University of California, Irvine, Irvine, CA 92697-2700 USA; 3 NIH Ultrasonic Transducer Resource Center, University of Southern California, Los Angeles, CA 90089 USA; 4 Shanghai Institute of Technology, 100 Haiquan Road, Fengxian Shanghai, China; 5 Division of Cardiology, Irvine Medical Center, University of California, Orange, CA 92868 USA

## Abstract

Cardiovascular diseases are the leading cause of fatalities in the United States. Atherosclerotic plaques are one of the primary complications that can lead to strokes and heart attacks if left untreated. It is essential to diagnose the disease early and distinguish vulnerable plaques from harmless ones. Many methods focus on the structural or molecular properties of plaques. Mechanical properties have been shown to change drastically when abnormalities develop in arterial tissue. We report the development of an acoustic radiation force optical coherence elastography (ARF-OCE) system that uses an integrated miniature ultrasound and optical coherence tomography (OCT) probe to map the relative elasticity of vascular tissues. We demonstrate the capability of the miniature probe to map the biomechanical properties in phantom and human cadaver carotid arteries.

## Introduction

Cardiovascular disease is the leading cause of death in the United States, resulting in every 1 in 3 deaths^1^. Atherosclerosis is a major underlying cause, in which plaque that is composed of fat, cholesterol, calcium, and other substances, build up in the artery walls. The lumen of the artery narrows and can lead to major diseases and complications, such as strokes and heart attacks. Identifying vulnerable plaques early is essential for the management and prevention of fatal outcomes^2^. Atherosclerotic plaques can occur anywhere in the major arterial vasculature, including smaller arteries such as the coronary artery that are not easily accessible^3^. Due to the lack of accessibility and the small size of the sample, intravascular diagnosis methods have become a powerful tool in interventional cardiology for diagnosis and management of cardiovascular diseases.

Several different intravascular imaging methods have been used to characterize the structure and properties of plaques and the vessel wall. Intravascular ultrasound (IVUS) is one of the oldest and most commonly used imaging modalities used to assess properties, such as the lumen size and wall morphology of arteries^4^. In more recent years, optical coherence tomography (OCT) is another intravascular imaging modality used to obtain high-resolution structural images^5^. More recently, the deep penetration of IVUS and high resolution of OCT have been combined in a single catheter to offer complementary structural information^6, 7^. However, structural information by itself is often not enough to make an accurate diagnosis on plaque vulnerability. Other methods, such as photoacoustic imaging and fluorescence detection have been used intravascularly to gain molecular and composition information of atherosclerotic plaques^8–12^.

Atherosclerosis also affects the mechanical properties of vessel walls due to the changes in the wall composition and the local geometry^13^. Since the biomechanical properties of fibrous and lipid plaques are different, there is a close correlation between the tissue elasticity and pathology. In recent years, several different elastography methods have been used to probe tissue elasticity. Acoustic radiation force has been vastly studied for ultrasound elastography, including acoustic radiation force impulse (ARFI) imaging, shear wave elasticity imaging, and vibro-acoustography^14–16^. These methods, especially ARFI, have been used in vascular tissue imaging and characterization of plaques. ARFI uses tissue displacement and relaxation information extracted from the response to a single impulse force and has been shown to characterize different types of plaques, and specifically carotid artery plaques *in vivo* ^17–19^. However, ARFI is limited by the resolution of ultrasound.

Optical coherence elastography (OCE) has the advantage of high resolution and high sensitivity for quantification of the mechanical structure of tissues^20–26^. OCE using dynamic excitation ARF has been studied for both shear wave and compressional wave generation^25–29^. However, these methods mostly focus on *ex vivo* image acquisition due to the size of the ultrasonic transducer and system stability. Taking the tissue out of its natural environment will affect its mechanical properties and have limited clinical value. In order to obtain accurate characterization of elastic properties, it is necessary to perform imaging *in vivo*, which requires incorporating the technique into a single miniature probe. Development of ARF-OCE with a miniature probe poses challenges due to the need for high sensitivity and a large excitation force.

In this paper, we introduce a novel miniature probe-based system using a single ARF as the excitation mechanism and phase-resolved OCE for detection in order to map the elasticity of tissues. We use a miniature focused ring transducer for maximum excitation and utilize OCE for its nanometer sensitivity^30–33^. Calibration data was obtained using two uniform phantoms while the feasibility of distinguishing mechanical contrast and tissue imaging was demonstrated using a side-by-side phantom and cadaver tissues, respectively. The relative Young’s moduli ratio of the cadaver tissue components were approximated and compared to literature values^34^. The results demonstrate the feasibility of a miniature probe for the quantification of tissue mechanical properties, and represent a significant first step toward developing an endoscopic intravascular probe for ARF-OCE.

## Results

### System Design

**Figure 1:**
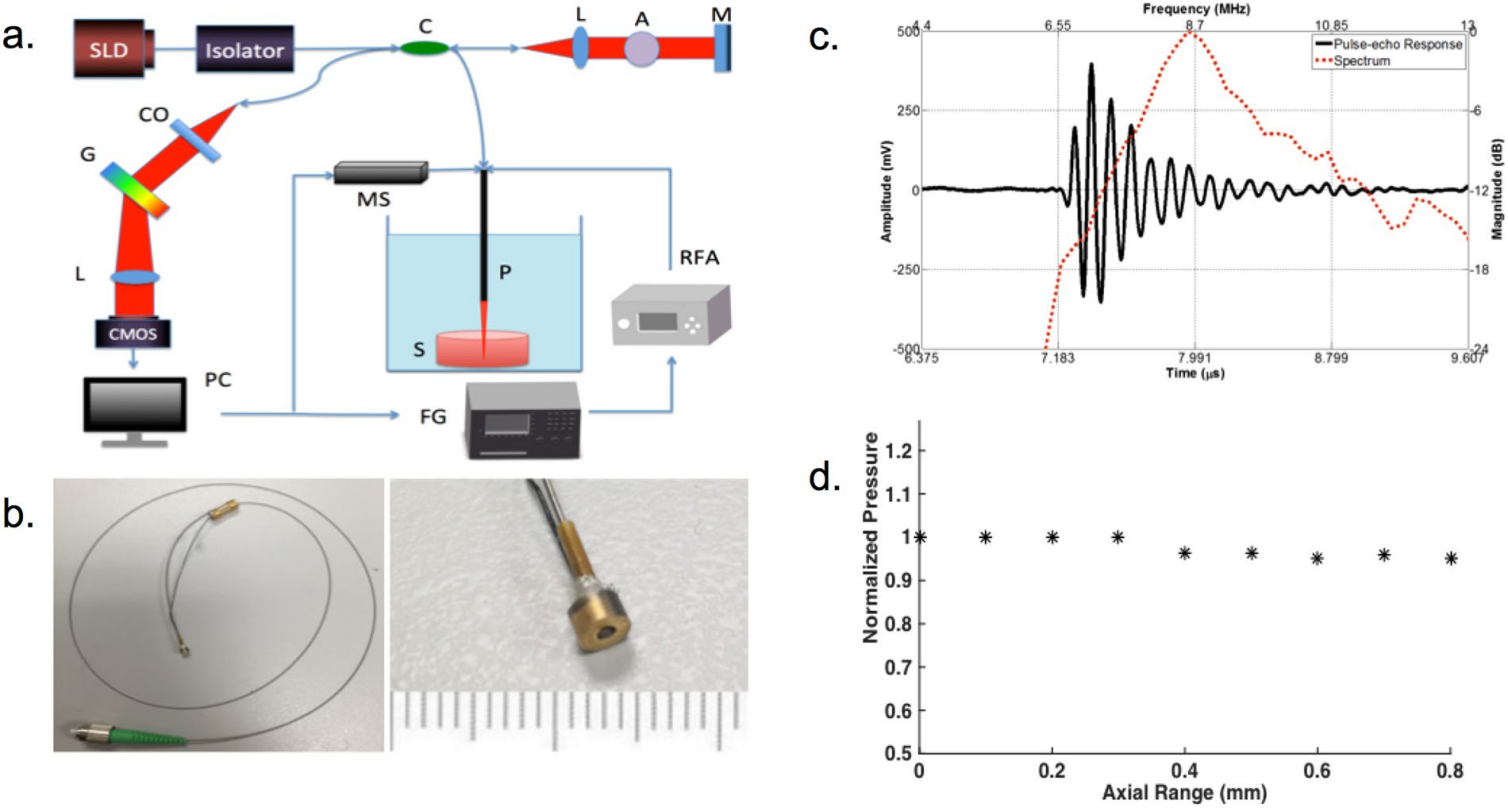
(**a**) Overview of ARFI OCE system set up. C: collimator, L: lens, A: attenuator, M: mirror, G: grating, MS: mechanical stage, RFA: radio frequency amplifier, P: probe, S: sample, FG: function generator. (**b**) Probe design (left). probe head including a ring transducer and optical elements inside (right). (**c**) Transducer frequency and echo characterization. (**d**) Transducer axial force field characterization.

The overall design of the ARFI-OCE system is shown in Fig. 1a. An 890 nm superluminescent diode (SLD) source with a bandwidth of 150 nm is used in the SD-OCT system. With a 0.9 mW output power on the sample, the system operates below the preset MPE limits. On the detection arm, a 1200 slits/mm diffraction grating is used along with a CMOS camera for detection of 20 k A-lines per second. The system has the capabilities to increase to 70 k A-line speed, but was not used for this feasibility study to maximize the signal to noise ratio. The ultrasonic excitation was synchronized with the OCE acquisition via a function generator for efficient detection. A single amplified ARFI was given for the specified pulse duration and 500 A-lines of Doppler OCT were used to detect the phantom or tissue response.

The front-facing miniature probe design is shown in Fig. 1b. A 3.5 mm 8.8 MHz ring ultrasound transducer with a middle aperture of approximately 1 mm with a focal depth of 5.53 mm was used. The optical components include HP-780 optical fiber and 0.7 mm in diameter GRIN lens. First, the single mode optical fiber is cleaved at 8 degrees using an angled fiber cleaver. Next, the GRIN lens is fixed to the fiber with UV glue. The focal distance of the fiber is kept at 5–6 mm by controlling for the distance between the lens and fiber end. A 0.8 mm in diameter polyimide tube is fastened over the lens-fiber complex for protection, and the entire unit is put through the aperture of the transducer. The surface of the transducer and lens are aligned flush so that excitation and detection are exactly confocal, yielding in maximum tissue response signal. The optical fiber is well protected inside a stainless steel torque coil housing and connects the imaging probe to the system via a FC/APC optical connector. One end of the fiber is connected to the GRIN lens for imaging, while the other end is connected to an FC/APC connector and attached to the system for light transmission.

The transducer characterization data including the pulse-echo response and the spectrum is shown in Fig. 1c. The electrical wire of the transducer connects to the function generator (FG) and radio frequency amplifier (RFA) for synchronization and driving ARF transducer. The tip of the probe was fixed to be stiff with glue and it was then fastened to a metal rod on the mechanical stage for stability and translational movement. The tip of the probe was submerged in a water bath to provide a medium for ultrasound during imaging. The mechanical stage movement was also synchronized with the ARFI excitation and Doppler OCT detection for scanning a B-scan. For calibration studies, only M-mode was used to capture the displacement response and the mechanical stage was not used for lateral scanning. The axial pressure field of the transducer is characterized in Fig. 1d. For this study, the sample placement was kept within 0.8 mm of the axial focal depth, where the excitation pressure is approximately uniform within a −0.4 dB range. Therefore, the displacement amplitude can be directly compared.

### Phase and Displacement Data

**Figure 2:**
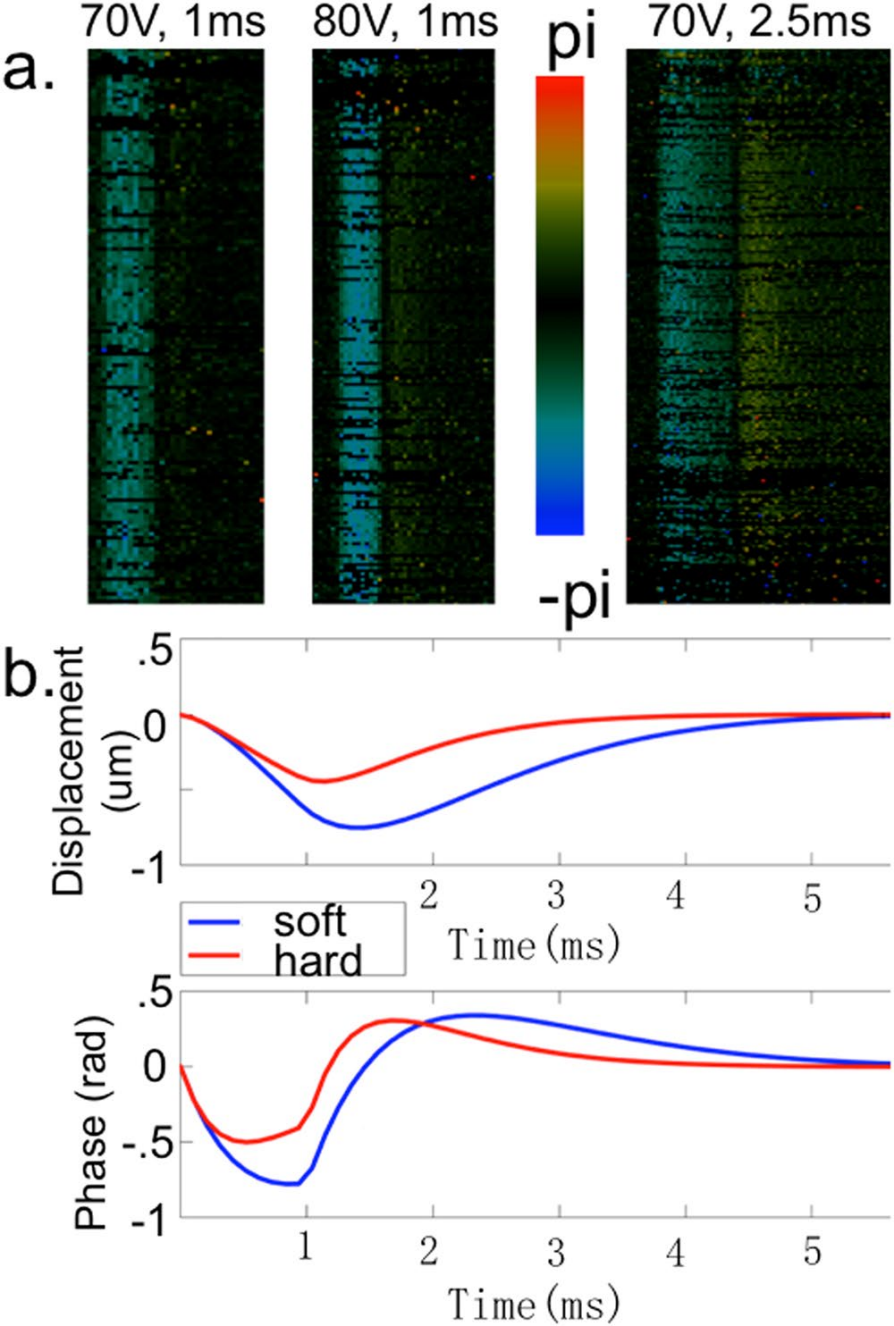
Displacement data. (**a**) Raw phase Doppler data of phantom response. (**b**) Displacement and phase analysis of response of 70 V, 1ms excitation.

Sample OCE raw images obtained from uniform gelatin phantoms are shown below. The left image in Fig. 2a, which shows the phase bands with a strong initial compression response and weaker relaxation response, was obtained using 70 V ARF excitation for the duration of 1 ms on a 12.7 kPa gelatin phantom. The middle image utilizes the same duration, but with a higher voltage of 80 V, and it is apparent that there is a difference in the phase bands, with the color changing from green to teal. In the right image, the pulse duration of the ARF is increased to 2.5 ms, and the phase bands for both the compression and relaxation become wider by approximately 2.5 fold.

In order to process and quantify the phase and displacement information, the phase Doppler equation (1) is used. The change in displacement, denoted by Δd, is calculated from the integral of the velocity, v. Using the concepts of the Doppler effect, the velocity can be obtained by calculating the change in phase information, or Δϕ(z,t). The central wavelength of the system is represented by λ_0_, the refractive index is n, the exposure time is τ, and the Doppler angle is θ. The displacement along the same direction as the excitation beam is given by:

1 $${\rm{\Delta }}d=\int v\,dt=\int \frac{{\rm{\Delta }}\varphi (z,t){\lambda }_{0}}{4\pi n\tau \,{\rm{c}}{\rm{o}}{\rm{s}}\theta }dt$$

Because the probe is very small and imaging takes place in a water bath, there is a need to eliminate the bulk motion in displacement caused by water movement and large-scale vibrations. Since the frequency of the noise is relatively low compared to that of the tissue response to the ARF, it is possible to use a polynomial regression fit to filter out the low frequency motions^35^. A 10^th^ order polynomial fit was used, based on least-squares regression analysis. This low frequency noise component was subtracted from the full signal so that only useful signal remained. The filter was applied on all displacement data to eliminate bulk motion.

In Fig. 2b, the response of a relatively soft and a relatively stiff gelatin phantom were obtained using a 70 V and 1 ms excitation ARF and both the phase and integrated displacement data are plotted. From the displacement image, it is apparent that the softer phantom displaced more than the stiffer phantom in general. For this particular data point, the maximum displacement of the soft phantom was 0.75 um while that of the stiff phantom was 0.45 um. The relaxation time for the soft phantom was also much longer than that of the stiff one, as expected. The phase plot shows the velocity of the soft phantom to be higher than that of the stiff one during the entire period.

### Phantom Studies

**Figure 3:**
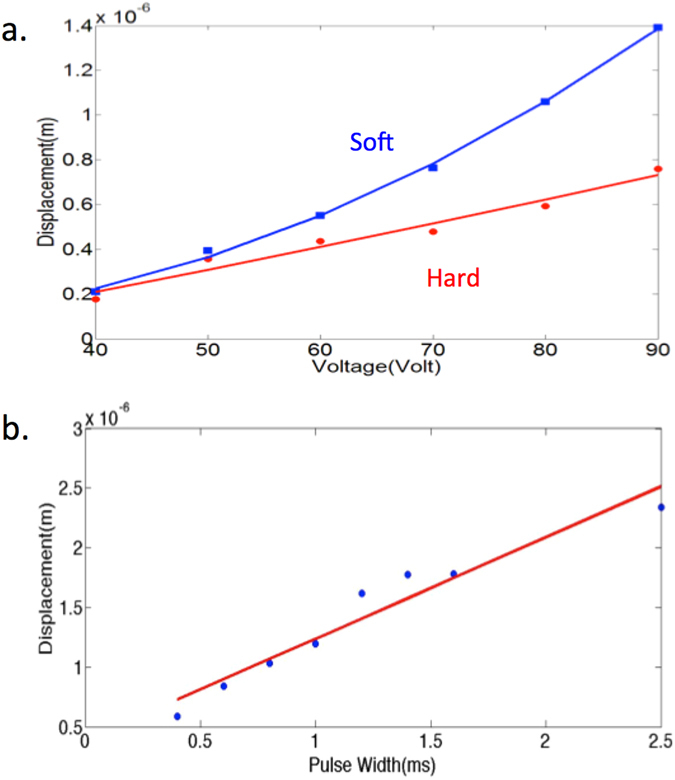
Phantom data. (**a**) Voltage versus displacement for relatively soft and stiff uniform gelatin phantoms. (**b**) Pulse width versus displacement for soft gelatin phantom.

Next, imaging was performed using 2 uniform gelatin phantoms. In Fig. 3a, various after-amplification voltages ranging from 50 V to 90 V were used on both phantoms and the displacement values were measured and averaged across 50 points. Bulk motion was once again removed. The relationship between the voltage and displacement is known to be quadratic, which is reflected in the regression fit in Fig. 3a. The soft phantom exhibited approximately 2 times the displacement value of the stiff one at each excitation voltage. Mechanical testing on the two phantoms was then performed, with the MTS Synergie 100 compression tester. The Young’s modulus of the soft phantom was 11.7 kPa while the Young’s modulus of the stiff phantom was 23.1 kPa. These results are in general agreement with the experimental data shown in Fig. 3a.

Data was also obtained for different excitation pulse widths. The soft gelatin phantom was used with pulse widths ranging from 0.4 to 2.5 ms. There is an approximately linear relationship between the pulse duration and the observed displacement as expected.

For the lateral scanning experiments, an excitation voltage of 70 V and a pulse width of 1 ms was used. The relationship between the Young’s modulus, Y, and sample displacement, ${\rm{\Delta }}d$, can be summarized using equation (2):

2 $$Y=\frac{\sigma }{{\rm{\Delta }}d/z}$$

where $\sigma$ is the stress induced by the ARF and z is the total thickness of the sample. Since the Young’s modulus for the soft and stiff phantoms were determined to be 11.7 kPa and 23.1 kPa respectively, corresponding to a soft to stiff elastic ratio to approximately 1:1.97. Since displacement and Young’s modulus is inversely proportional, this displacement of the soft to stiff phantom is expected to be 1.97:1, which is approximately the ratio denoted in Fig. 3a.

### Lateral Scanning

#### Side-by-side Phantom

**Figure 4:**
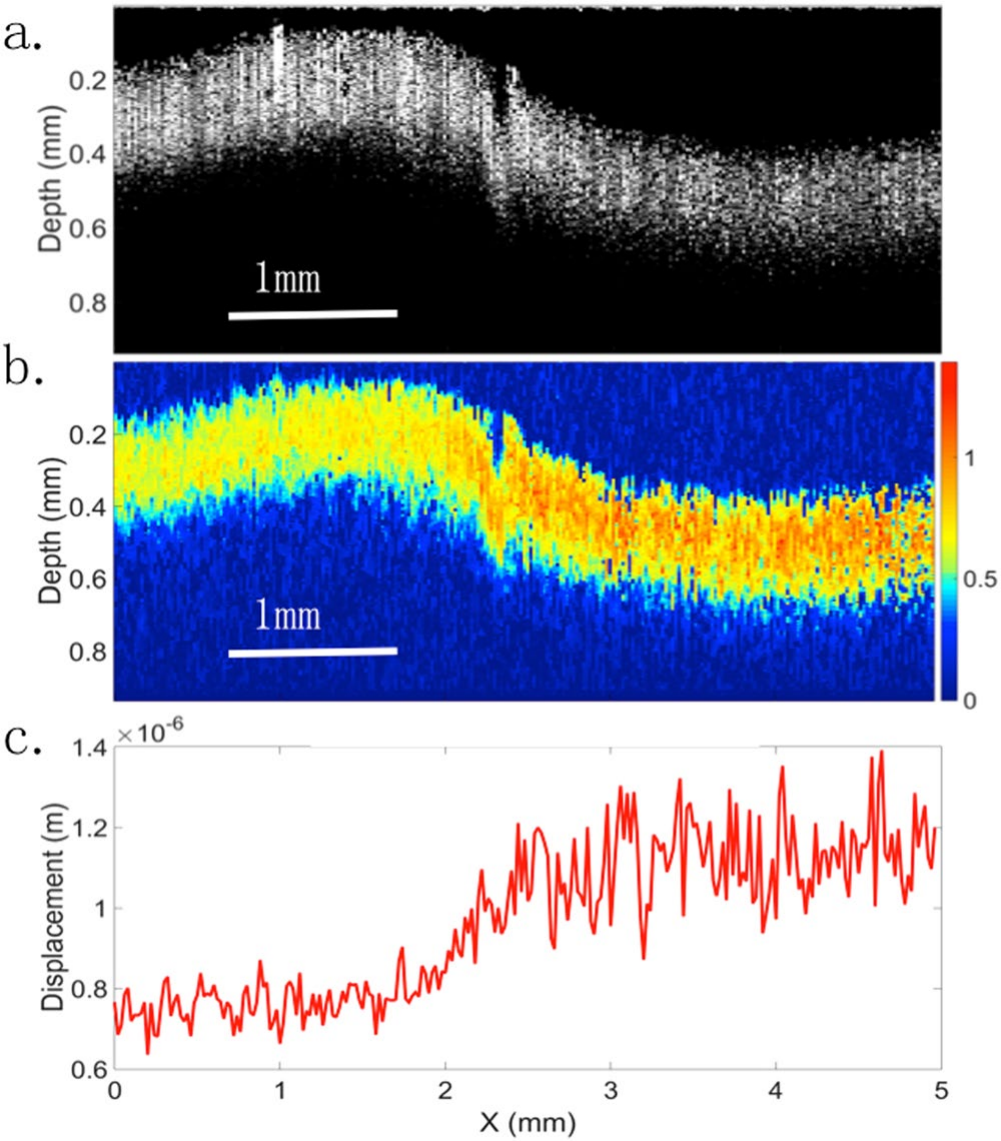
Side by side phantom. (**a**) OCT structural image. (**b**) OCE elastogram. (**c**) Average displacement map.

Next, in order to validate the feasibility of lateral scanning using a mechanical stage without excess noise, a side-by-side gelatin phantom was made in a 5 cm diameter petri dish with a height of 1 cm, and placed upright at the bottom of the water bath for imaging. The mechanical stage was synchronized with a step size of 20 μm, traveling one step every second. At each step, a 70 V and 1 ms excitation pulse was given, and 500 A-lines were recorded to capture the sample response. The structural OCT image is shown in Fig. 4a. The 2-sided phantom appears uneven within the ultrasound focal region, with the right surface at a lower position than the left side due to a few different factors, including initial fabrication artifact, diffusion, and evaporation of the phantom between its construction and the time of imaging. This effect has been noted and so mechanical testing was performed immediately after imaging to minimize any changes in the elasticity over time. The OCT image shows a relatively uniform structure throughout. In Fig. 4b, the elastogram of the same phantom is shown, and it is apparent that the right hand side shows higher displacement than the left side. Figure 4c draws out the average displacement curve by using segmentation to isolate the entire signal region in Fig. 4b and taking the mean along the entire depth. The left side has mean value of 0.75 μm while the right side has a mean of 1.13 μm. The displacement values are larger than that of the homogeneous phantoms since the thickness of the side-by-side phantom was larger, at 8 mm. There is a gradual gradient change in the middle region where the two phantom likely diffused and mixed with each other.

#### Human Cadaver Carotid Artery

**Figure 5:**
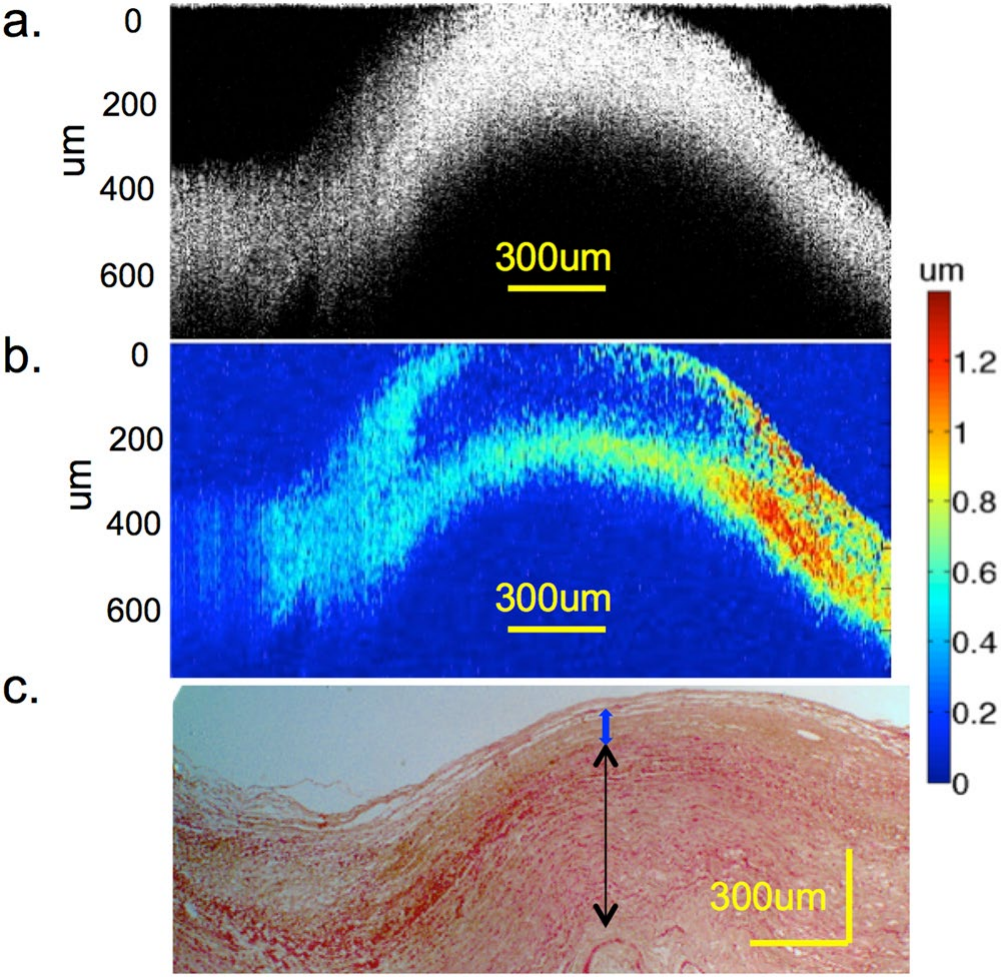
Cadaver tissue & histology data. (**a**) OCT image of human cadaver coronary artery cross-section. (**b**) OCE elastogram of corresponding region. **(c**) H&E staining of the region under 4x magnification. Red box corresponds to region of stiff inclusion.

Finally, the carotid artery of a human cadaver was used to test the system response in tissue, specifically in plaque regions. The images were taken with similar parameters as that of the side-by-side phantom, at 20 Hz A-line rate in M-mode, with 1 s per movement step on the mechanical stage. A post-amplified voltage of 70 V and pulse duration of 1ms was used. The OCT image is shown in Fig. 5a, where it is difficult to distinguish the plaque tissue from just the structural data. There seems to be a structural change on the left hand side of the image that may correspond to a plaque. However, the right hand side looks to be relatively uniform and smooth.

From the OCE elastogram shown in Fig. 5b, it is apparent that the displacement on the left hand side is much smaller than the right hand side, suggesting plaque formation there. More interestingly, within the middle bulging section, there are distinct layers on the tissue. The dark blue region is much stiffer, suggesting that there may be a fibrous plaque layer there. The right hand side of the image shows higher displacement and signals toward relatively healthy tissue.

In order to validate the feasibility of our system in discerning abnormalities in tissue, histology was performed on the matching tissue using H&E staining shown in Fig. 5c. A research pathologist was consulted in reading the histological slide. There is relative preservation of the smooth muscle layers in the media of the artery on the right hand portion, shown by the black arrow. However, there is obvious intimal thickening and atheroma on the lumen side, indicated by the blue arrow, which correspond to the low displacement region in the middle of Fig. 5b. Within the 300 um tissue penetration that was imaged, the intimal thickening was fully captured, shown by the yellow layer. On the left hand side, there is a thicker atherosclerotic plaque, for which the vacuoles can be visualized, and this can be correlated to the lower displacement on the left side than the right side. Based on the morphology, these indications are consistent with atherosclerotic plaques. This demonstrates that the mechanical elasticity seen in our elastogram matches well with tissue pathology. It is also important to note that the system has the capabilities to differentiate both lateral and axial contrast within arterial tissue. A gradient can be seen from the left side where it is stiffer to the right side where the healthier tissue is soft. This can potentially allow us to diagnose the severity of stiffening and disease progression.

Using the calibration data for the ARF stress value, we can approximate the relative Young’s moduli of the healthy versus diseased region. In Fig. 5 above, the far right side consists of mostly healthy tissue and early atheroma, and the average displacement is estimated to be 0.97 ± 0.2 um. The left side, consisting of abnormal tissue, has a displacement of 0.19 ± 0.04 um. This corresponds to a stiffness ratio of 1:5.1 for the right to left sides. According to this value and comparison to literature^34^, the diseased region is most likely composed of fibrous plaque, which has been verified with histology as well.

## Discussion and Conclusion

Although we demonstrated that we can obtain ARF-OCE imaging with a miniature ARF transducer, there are a few limitations that need to be overcome before this technology can be translated to *in vivo* intravascular imaging. Current imaging speed is too slow, at approximately 8 minutes per 2D scan, and continuous excitation may be necessary to increase the imaging speed. In addition, the prototype probe is currently 3.5 mm in diameter, which is limited by the ring ultrasound transducer. In order to perform intravascular imaging especially in the smaller coronary arteries, the transducer needs to be further reduced to about 1 mm. Furthermore, a side-facing probe must be implemented. We have previously demonstrated integrated OCT/US probes with a diameter of less than 1 mm, and translated this technology for *in vivo* imaging^6^. Another issue to consider is the bulk motion of the ARF in *in vivo* studies, where the probe will not be supported by a metal rod. Further tests need to be performed in order to validate the effect of such noise. In addition, the cadaver sample used in this experiment only had fibrous plaque, and did not have any lipid or calcifications. Since the acoustic properties of fibrous plaque is similar to that of smooth muscle in the artery, the displacement amplitude could be used alone for relative stiffness estimations^36^. However, since the properties are significantly different in lipid^37^, additional tests and parameters, such as the time to peak displacement and recovery time, may be used to characterize the mechanical properties. Finally, current system uses an OCT system centered at 890 nm wavelength, which limits the imaging depth. The penetration range can be improved by either using a 1.3 um center wavelength source in the system or increasing the output power.

In conclusion, we have introduced an ARFI-OCE technique that uses a front-facing miniature probe with a ring transducer to generate elastograms of tissue with high mechanical sensitivity. Phantom data was collected using two uniform phantoms with different stiffness. Lateral scanning was performed using a side-by-side gelatin phantom and observed the appropriate mechanical contrast. Finally, imaging was performed on a human cadaver carotid artery sample and tissue abnormality was detected and matched to histology results. The relative Young’s moduli of the tissue were approximated based on the relative displacement information. These results represent a prototype device that can identify plaque regions effectively and serves as an important step to the miniaturization and translation of this technology for *in vivo* imaging.

## Methods

### Phantom preparation

Two gelatin phantoms with different stiffness were fabricated. The relatively soft phantom was composed for 2.45% (by weight) gelatin, 0.4% (by volume) intralipid, and 1.8% (by weight) silicon dioxide. The stiffer phantom composed of 6% (by weight) gelatin. Intralipid was added for optical contrast while silicon dioxide was for acoustic attenuation. The uniform phantoms are circular with a diameter of 5 cm and thickness of 5 mm. A side-by-side phantom was also fabricated by cutting out a portion of the stiff phantom and adding the gelatin mixture of the soft phantom to the opening. The thickness of the side-by-side phantom was approximately 8 mm. There was some diffusion that occurred between the barriers of the two phantoms, which contributed to a gradient in elasticity.

### Cadaver tissue preparation

Fresh human carotid artery samples were obtained from volunteer subjects and frozen in a −19 C degree freezer. After the imaging system was set up, the tissue was thawed and a 1 cm section of the tissue was isolated and cut open longitudinally. The tissue was pinned down to a holder and submerged in phosphate buffered saline for imaging to minimize swelling. After imaging was completed, the region of interest was marked with pins and the tissue was fixed in formalin for 24 hours. Then it was processed and embedded in wax. Sectioning was performed using a Microtome to obtain 6 um thick slices on a region of interest of 1mm. Finally, H&E staining was performed and images were taken with a microscope with 4x magnification to find a match with the experimental region of interest.

All methods were carried out in accordance with the University of California, Irvine (UCI) Institutional Review Board (IRB) and the Institutional Biosafety Committee (IBC). IRB granted an exemption to the protocol requirement since the activities do not constitute Human Subject Research. Informed consent was deemed unnecessary because confidentiality of the deceased cadaver tissues is protected and coded. All experimental protocols were approved by the UCI IBC under protocol #2016–1570.

### Compression Testing

Mechanical compression tests were performed on the 2 uniform gelatin phantoms using a MTS Synergie 100. A strain of up to 0.1 mm/mm was used, with a strain rate of 50 mm/min. The thickness of the tissue was approximately 1 cm, and 50% compression was used. In most cases, deterioration was seen at around 50%.

## References

[1] Mozaffarian D (2015). Heart disease and stroke statistics-2015 update: a report from the. American heart association. Circulation.

[2] Puri R, Worthley MI, Nicholls SJ (2011). Intravascular imaging of vulnerable coronary plaque: Current and future concepts. Nature Rev. Cardiol..

[3] VanderLaan P, Reardon CA, Getz GS (2004). Site specificity of atherosclerosis. Arteriosclerosis, thrombosis, and vascular biology.

[4] Nissen SE (1991). Intravascular ultrasound assessment of lumen size and wall morphology in normal subjects and patients with coronary artery disease. Circulation.

[5] Bezerra HG, Costa MA, Guagliumi G, Rollins AM, Simon DI (2009). Intracoronary optical coherence tomography: a comprehensive review: clinical and research applications. JACC: Cardiovascular Interventions.

[6] Li X (2014). Integrated IVUS-OCT imaging for atherosclerotic plaque characterization. IEEE Journal of Selected Topics in Quantum Electronics.

[7] Li, J. *et al.* Ultrafast optical-ultrasonic system and miniaturized catheter for imaging and characterizing atherosclerotic plaques in vivo. *Scientific Reports* **5**, 18406 (2016). 10.1038/srep18406 PMC4683418 26678300

[8] Hui J (2015). High-speed intravascular photoacoustic imaging at 1.7 μm with a KTP-based OPO. Biomedical optics express.

[9] Liang S (2012). Intravascular atherosclerotic imaging with combined fluorescence and optical coherence tomography probe based on a double-clad fiber combiner. Journal of biomedical optics.

[10] Li Y (2016). Fully integrated optical coherence tomography, ultrasound, and indocyanine green-based fluorescence tri-modality system for intravascular imaging. Biomedical Optics Express.

[11] Li Y (2015). High-speed intravascular spectroscopic photoacoustic imaging at 1000 A-lines per second with a 0.9-mm diameter catheter. Journal of biomedical optics.

[12] Piao, Z. *et al*. High speed intravascular photoacoustic imaging with fast optical parametric oscillator laser at 1.7 um. A*pplied Physics Letters* **107**, 083701 (2015). 10.1063/1.4929584 PMC4552696 26339072

[13] Richardson, P. D. In *Mechanical properties of atherosclerotic tissues*. (Springer Berlin Heidelberg, 2006).

[14] Doherty JR, Trahey GE, Nightingale KR, Palmeri ML (2013). Acoustic radiation force elasticity imaging in diagnostic ultrasound. IEEE transactions on ultrasonics, ferroelectrics, and frequency control.

[15] Fatemi M, Greenleaf JF (1999). Vibro-acoustography: An imaging modality based on ultrasound-stimulated acoustic emission. Proceedings of the National Academy of Sciences.

[16] Nightingale K, Soo MS, Nightingale R, Trahey G (2002). Acoustic radiation force impulse imaging: *in vivo* demonstration of clinical feasibility. Ultrasound in medicine & biology.

[17] Dahl JJ, Dumont DM, Allen JD, Miller EM, Trahey GE (2009). Acoustic radiation force impulse imaging for noninvasive characterization of carotid artery atherosclerotic plaques: a feasibility study. Ultrasound in medicine & biology.

[18] Trahey GE, Palmeri ML, Bentley RC, Nightingale KR (2004). Acoustic radiation force impulse imaging of the mechanical properties of arteries: *in vivo* and *ex vivo* results. Ultrasound in medicine & biology.

[19] Allen, J. D. *et al*. The development and potential of acoustic radiation force impulse (ARFI) imaging for carotid artery plaque characterization. *Vascular Medicine* 1358863X11400936 (2011). 10.1177/1358863X11400936 PMC3265036 21447606

[20] Zhu, J. *et al*. Imaging and characterizing shear wave and shear modulus under orthogonal acoustic radiation force excitation using OCT Doppler variance method. *Optics Letters* **40**, (2015). 10.1364/OL.40.002099 PMC4537318 25927794

[21] Zhu J (2017). Longitudinal shear wave imaging for elasticity mapping using optical coherence elastography. Applied Physics Letters.

[22] Wang S, Larin KV (2014). Shear wave imaging optical coherence tomography (SWI-OCT) for ocular tissue biomechanics. Optics Letters.

[23] Kennedy BF, Kennedy KM, Sampson DD (2014). Review of Optical Coherence Elastography Fundamentals, Techniques, and Prospects. IEEE Journal of Selected Topics in Quantum Electronics.

[24] Xu X, Zhu J, Chen Z (2016). Dynamic and quantitative assessment of blood coagulation using optical coherence elastography. Nature Scientific Reports.

[25] Qi W (2013). Resonant acoustic radiation force optical coherence elastography. Applied Physics Letters.

[26] Qi W (2012). Phase-resolved acoustic radiation force optical coherence elastography. Journal of biomedical optics.

[27] Sarvazyan A (2011). An overview of elastography – an emerging branch of medical imaging. Curr. Med. Imaging Rev..

[28] Qu Y (2016). “Acoustic Radiation Force Optical Coherence Elastography of Corneal Tissue.”. Selected Topics in Quantum Electronics, IEEE Journal of.

[29] Zhu, J. *et al*. “3D mapping of elastic modulus using shear wave optical micro-elastography.” *Scientific Reports* 6, doi:10.1038/srep35499 (2016). 10.1038/srep35499 PMC5071855 27762276

[30] Zhao Y, Chen Z, Saxer C, Xiang S, de Boer JF, Nelson JS (2000). Phase-resolved optical coherence tomography and optical Doppler tomography for imaging blood flow in human skin with fast scanning speed and high velocity sensitivity. Optics Letters.

[31] Joo C, Akkin T, Cense B, Park BH, de Boer JF (2005). Spectral-domain optical coherence phase microscopy for quantitative phase-contrast imaging. Optics Letters.

[32] Choma MA, Ellerbee AK, Yang C, Creazzo TL, Izatt JA (2005). Spectral-domain phase microscopy. Optics Letters.

[33] Zhang, J., Rao, B., Yu, L. & Chen, Z. High-dynamic-range quantitative phase imaging with spectral domain phase microscopy. *Optics Letters*, (2009). 10.1364/OL.34.003442 PMC3337213 19881621

[34] Tracqui P (2011). Mapping elasticity moduli of atherosclerotic plaque *in situ* via atomic force microscopy. Journal of Structural Biology.

[35] Kadi AP, Loupas T (1995). On the performance of regression and step-initialized IIR clutter filters for color Doppler systems in diagnostic medical ultrasound. IEEE transactions on ultrasonics, ferroelectrics, and frequency control.

[36] Picano E (1985). Fibrosis, lipids, and calcium in human atherosclerotic plaque. In vitro differentiation from normal aortic walls by ultrasonic attenuation. Circulation research.

[37] Wang, X., Salamat, M. S., Varghese, T. & Dempsey, R.J. Carotid plaque characterization with histology and quantitative ultrasound. *Ultrasonics Symposium (IUS)*, **2014** *IEEE International*, 2386–2389 (2014).

